# Identification of crucial anoikis-related genes as novel biomarkers and potential therapeutic targets for lung adenocarcinoma via bioinformatic analysis and experimental verification

**DOI:** 10.18632/aging.205521

**Published:** 2024-02-09

**Authors:** Jie Wu, Yuting Zhang, Guoxing You, Wenjie Guo, Yupeng Wang, Jiaming Li, Rongzhi Tan, Xihua Fu, Yukuan Tang, Jie Zan, Jianfen Su

**Affiliations:** 1 Department of Pharmacy, Guangzhou Panyu Central Hospital, Guangzhou 511400, China; 2 School of Biomedical and Pharmaceutical Sciences, Guangdong University of Technology, Guangzhou 510006, China; 3 The Second School of Clinical Medicine, Southern Medical University, Guangzhou 510515, China; 4 Department of Infectious Diseases and Hepatology Unit, Guangzhou Panyu Central Hospital, Guangzhou 511400, China; 5 Department of Minimally Invasive Interventional Radiology, Guangzhou Panyu Central Hospital, Guangzhou 511400, China

**Keywords:** lung adenocarcinoma, anoikis, stratified model, precision medication, immune microenvironment

## Abstract

Lung adenocarcinoma (LUAD) is a malignant tumor of the respiratory system that has a poor 5-year survival rate. Anoikis, a type of programmed cell death, contributes to tumor development and metastasis. The aim of this study was to develop an anoikis-based stratified model, and a multivariable-based nomogram for guiding clinical therapy for LUAD. Through differentially expressed analysis, univariate Cox, LASSO Cox regression, and random forest algorithm analysis, we established a 4 anoikis-related genes-based stratified model, and a multivariable-based nomogram, which could accurately predict the prognosis of LUAD patients in the TCGA and GEO databases, respectively. The low and high-risk score LUAD patients stratified by the model showed different tumor mutation burden, tumor microenvironment, gemcitabine sensitivity and immune checkpoint expressions. Through immunohistochemical analysis of clinical LUAD samples, we found that the 4 anoikis-related genes (PLK1, SLC2A1, ANGPTL4, CDKN3) were highly expressed in the tumor samples from clinical LUAD patients, and knockdown of these genes in LUAD cells by transfection with small interfering RNAs significantly inhibited LUAD cell proliferation and migration, and promoted anoikis. In conclusion, we developed an anoikis-based stratified model and a multivariable-based nomogram of LUAD, which could predict the survival of LUAD patients and guide clinical treatment.

## INTRODUCTION

Lung cancer is a malignant tumor with a high fatality rate worldwide [1]. About 85% of lung cancers are non-small cell lung cancers (NSCLC), and about 60% of NSCLCs are lung adenocarcinomas (LUAD). Nowadays, surgery, chemoradiotherapy, and targeted medication therapy are wildly used to treat lung cancer [2]. However, the therapeutic effect and the long-term survival rate of LUAD patients are still not ideal. Thus, identification of new prognostic markers for patients with LUAD is essential for early detection and precision medication.

When a cell separates from the nearby extracellular matrix (ECM), a form of programmed cell death known as anoikis begins [3]. Under normal conditions, anoikis removes displaced cells and prevents them from attaching improperly [4]. However, several malignant tumor metastases are closely associated with anoikis resistance which enables carcinoma cells to escape apoptosis and establish a metastatic lesion, including lung cancer [5], hepatocellular carcinoma [6], gastric cancer [7], gliomas, and so on [8]. Nowadays, several pathways and essential genes have been identified to be involved in anoikis resistance. For example, the PLAG1-GDH1 axis promotes anoikis resistance and tumor metastasis through CamKK2-AMPK signaling in LKB1-deficient lung cancer [5]. Nuclear MYH9-induced CTNNB1 transcription promotes gastric cancer cell anoikis resistance and metastasis [7]. SPIB promotes anoikis resistance via elevated autolysosomal process in lung cancer cells [9]. Thus, discovering essential anoikis genes and associated pathways in LUAD are critical for development of therapeutic drugs for LUAD.

In the current study, we explored the differentially expressed genes (DEGs) that are associated with anoikis in healthy and LUAD specimens, and developed a predictive risk score signature of anoikis in LUAD. This signature could predict the malignant degree and prognosis of LUAD patients and effectively guide clinical chemotherapy. The results of this study may provide a new strategy for exploring the treatment of LUAD.

## RESULTS

### Identification of anoikis-related DEGs (ARGs) in LUAD

**Figure 1:**
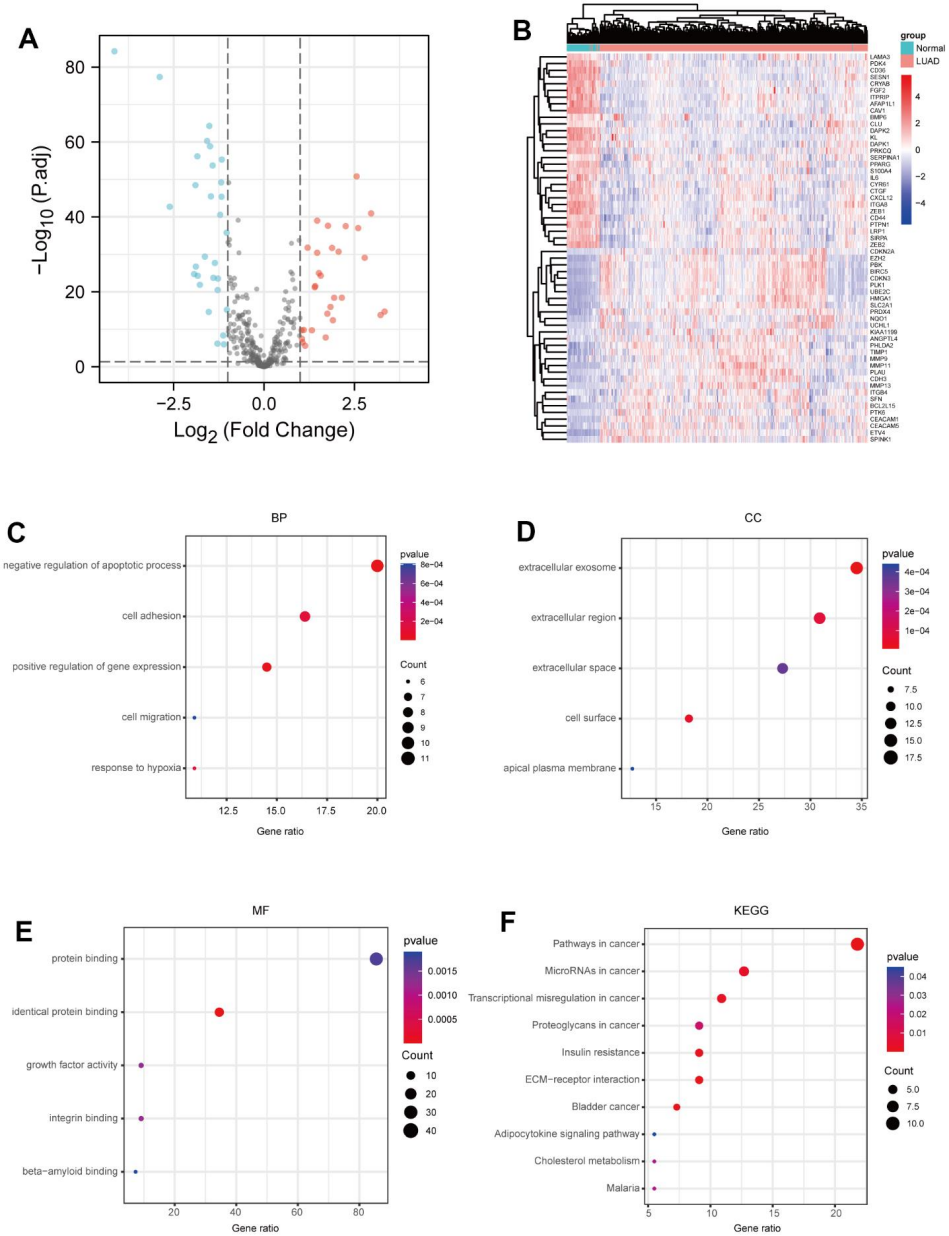
**Identification of ARGs in LUAD.** (**A**) Volcano plot anoikis-related genes in TCGA dataset. |LogFC|>1 and adj.P-value < 0.05 were set to screen. (**B**) Heatmap of the ARGs in TCGA dataset. (**C**) BP analysis of ARGs. (**D**) CC analysis of ARGs. (**E**) MF analysis of ARGs. (**F**) KEGG analysis of ARGs. P-value <0.05 is considered significant.

Firstly, we analyzed the expressions of ARGs in LUAD using the mRNA profiles of 456 samples from the TCGA-LUAD dataset (https://portal.gdc.cancer.gov/), and identified 29 up-regulated ARGs and 29 down-regulated ARGs in LUAD samples, compared to normal samples (Figure 1A, 1B and Supplementary Table 1). Next, we further investigated the biological functions of the identified ARGs in LUAD through Gene Ontology (GO) annotations and Kyoto Encyclopedia of Genes and Genomes (KEGG) enrichment analysis. As shown in Figure 1C, negative regulation of apoptotic process, positive regulation of cell migration and cell-matrix adhesion were enriched in the biological process (BP) category. Extracellular exosome, extracellular region and extracellular surface were enriched in the cellular component (CC) category (Figure 1D). Protein binding, identical protein binding and growth factor activity were enriched in the molecular function (MF) category (Figure 1E). Furthermore, KEGG pathway enrichment analysis showed that these identified ARGs were mainly enriched in the pathways in cancer, microRNAs in cancer and transcriptional misregulation in cancer (Figure 1F).

### Construction of a prognostic signature based on ARGs

**Figure 2:**
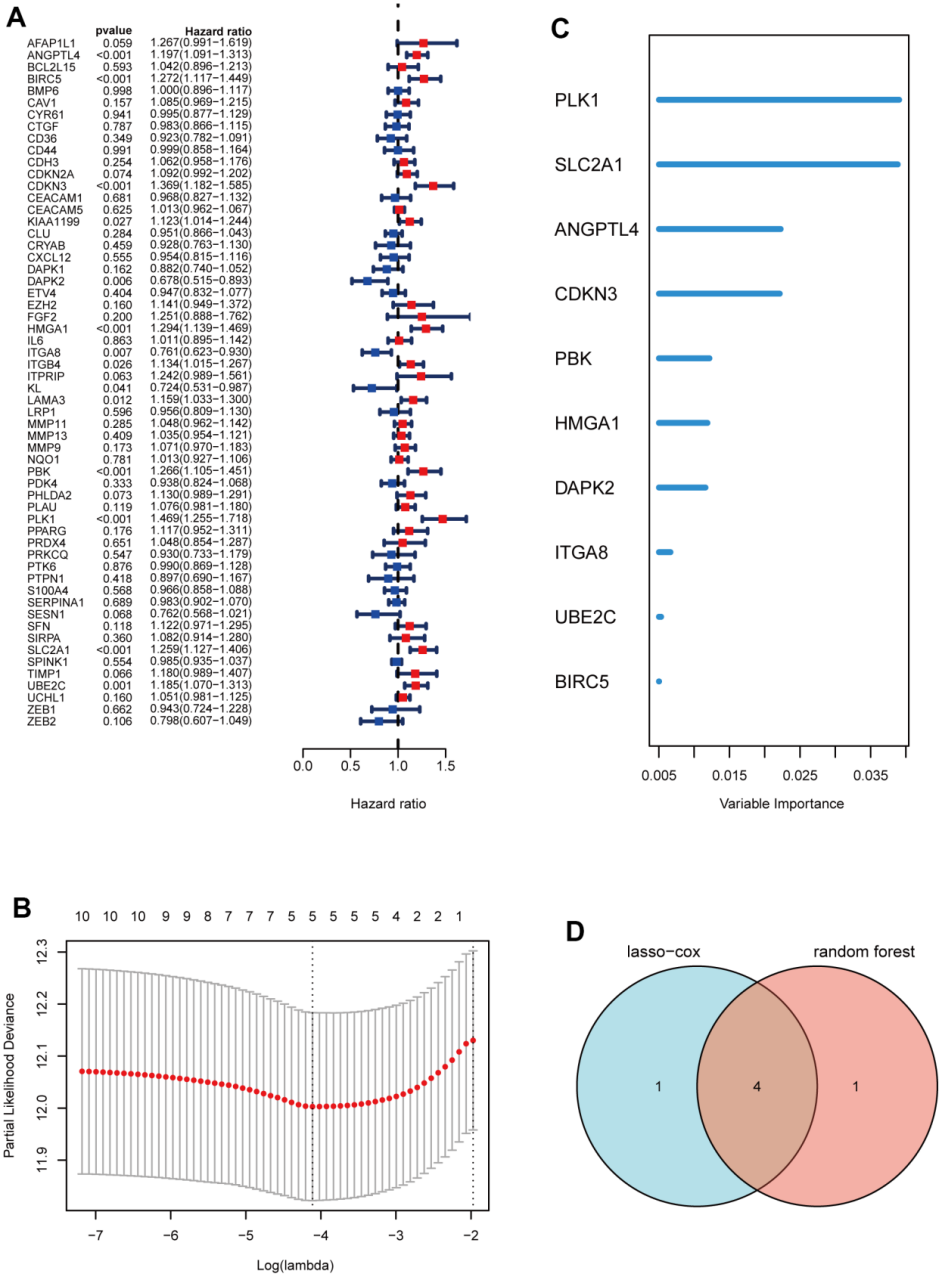
**Construction of a prognostic signature based on ARGs.** (**A**) Forest maps of univariate Cox analysis. (**B**) a minimum value of λ was chosen as optimal. The black dot line on the left represents those 10 features that were reduced to 5 non-zero coefficient features by LASSO. (**C**) ARGs rank importance by random forest algorithm. (**D**) Venn diagram shows the intersection ARGs of Lasso and random forest algorithm.

**Figure 3:**
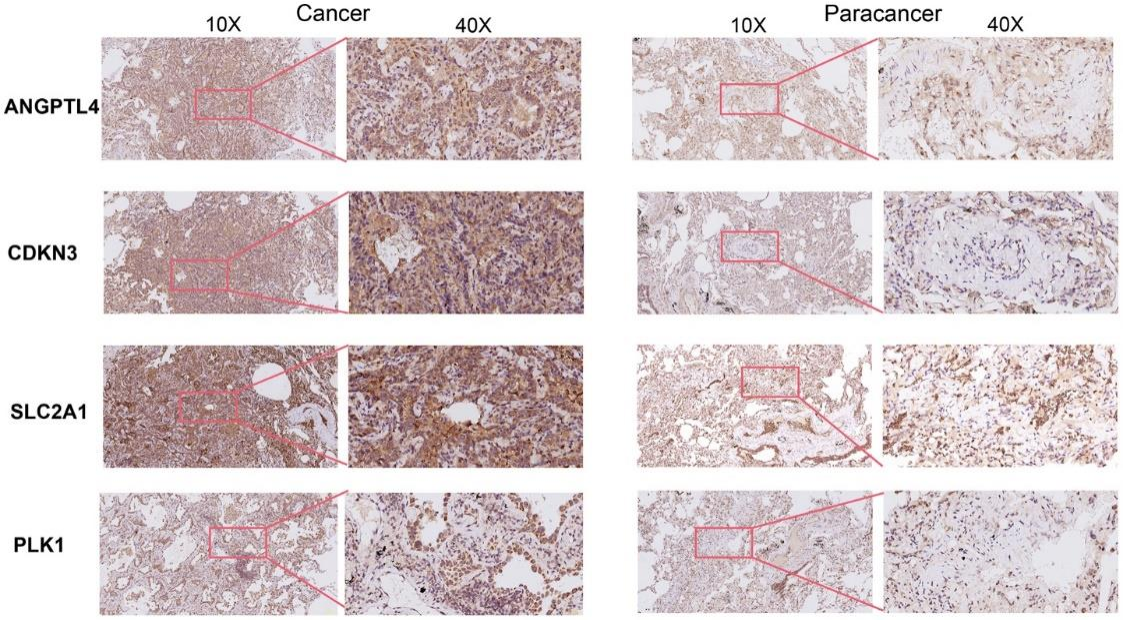
Immunohistochemical staining of four ARGs in cancer tissues and para-cancer tissues from clinical LUAD patients.

Subsequently, through univariate Cox analysis of the aforementioned 58 ARGs, 10 genes (PLK1, SLC2A1, ANGPTL4, CDKN3, PBK, HMGA1, DAPK2, ITGA8, UBE2C, and BIRC5) were identified to be linked with LUAD prognosis (p<0.01) (Figure 2A and Supplementary Table 2). Then, we performed the least absolute shrinkage and selection operator (LASSO) Cox regression analysis to avoid excessive variables [10], and identified five ARGs (PLK1, SLC2A1, ANGPTL4, CDKN3, HMGA1) (Figure 2B). Meanwhile, we performed the random forest algorithm to rank the importance of ARGs [11], and selected the top five important ARGs (PLK1, SLC2A1, ANGPTL4, CDKN3, PBK) (Figure 2C). Furthermore, four ARGs (PLK1, SLC2A1, ANGPTL4, CDKN3) were obtained by intersections of ARGs screened by the above two machine learning algorithms (Figure 2D), and these four genes have been found to play essential roles in LUAD development [12–15]. Moreover, we examined the protein expression levels of the four ARGs (PLK1, SLC2A1, ANGPTL4, CDKN3) in cancer tissues and para-cancer tissues from clinical LUAD patients. As shown in Figure 3 and Supplementary Figure 1, the protein expression levels of PLK1, SLC2A1, ANGPTL4, and CDKN3 in LUAD tissues were all significantly higher than those in para-cancer tissues. Finally, we created a prognostic risk signature using the above four genes via multivariate Cox regression, and the formula is as: riskscore = 0.13445 * Exp (ANGPTL4) + 0.12767 * Exp (CDKN3) + 0.21102 * Exp (PLK1) + 0.042861 * Exp (SLC2A1).

### Evaluation of the 4 ARGs-based prognostic signature

**Figure 4:**
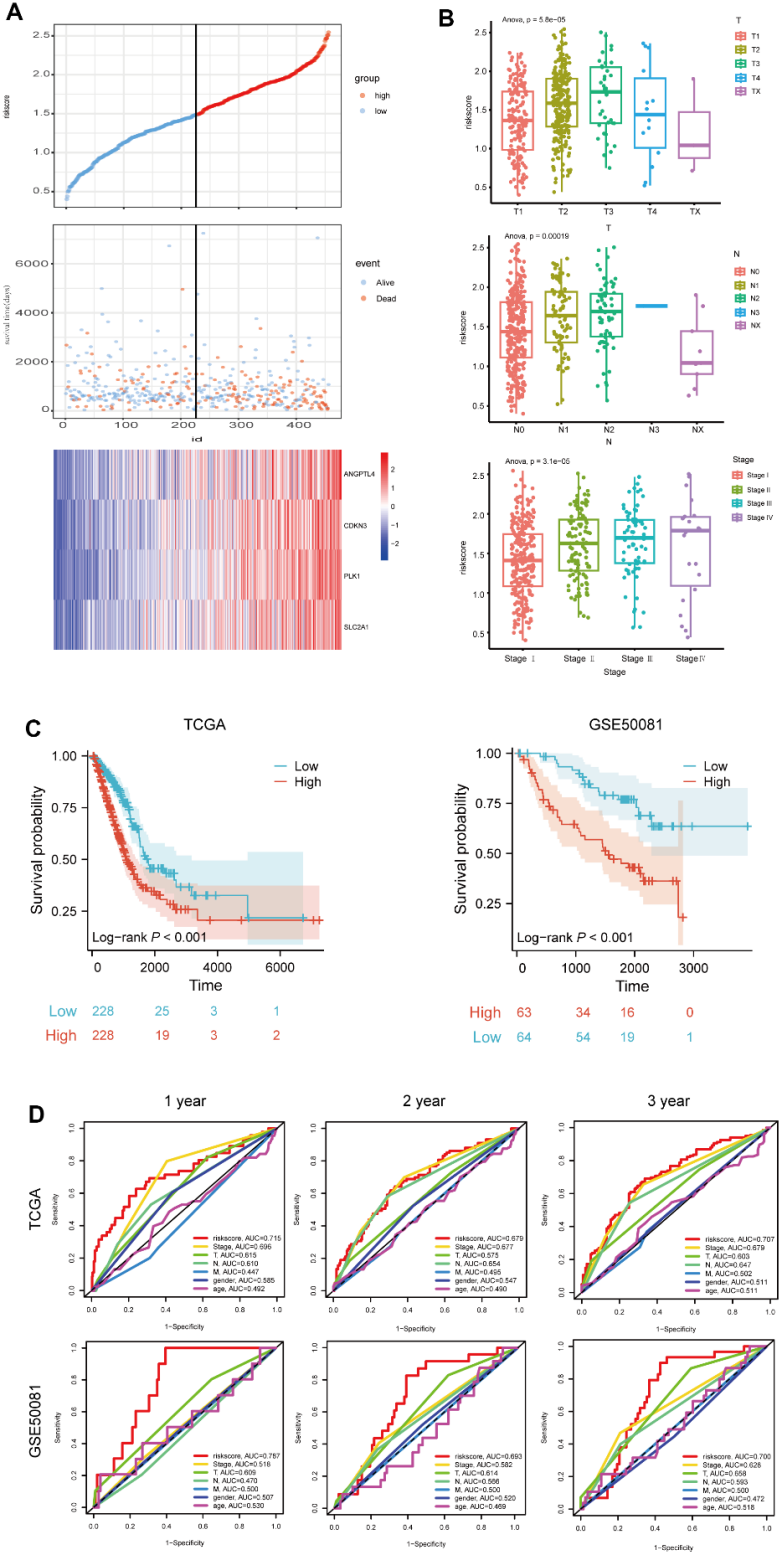
**Evaluation of the 4 ARGs-based prognostic signature.** (**A**) The distribution of risk score, patients' survival and status for LUAD. The black dotted line divided patients into high-risk group and low-risk group. (**B**) The box plot shows the relationship between riskscore and T, N and stage in TCGA. (**C**) Kaplan-Meier survival analysis of patients stratified by the median risk score in TCGA and GSE50081. (**D**) The ROC curve was applied to compare the predictive power of riskscore and clinical features in TCGA and GSE50081.

To further evaluate the prognostic risk signature, LUAD patients from the TCGA dataset were stratified into two groups based on the median risk score, and the high- risk score group had a shorter survival time, but had higher expressions of the above four genes (Figure 4A). Furthermore, we analyzed the correlation between risk score and clinicopathological characteristics of LUAD patients, and found that the levels of T, N, and stage of LUAD patients rose with risk score (Figure 4B). Moreover, through survival probability analysis using the model in the LUAD samples of the TCGA dataset or GSE50081 which were used as a validation set, we found LUAD patients with high-risk score had a bad prognosis (log-rank p<0.001) (Figure 4C). In addition, we assessed the risk score's predictive value using the ROC analysis, and found that risk score had the largest AUC area, compared to stage, T, N, M, gender, or age (Figure 4D). Collectively, these results suggest the good prognostic performance of our model.

### Developing a nomogram for predicting LUAD patients' survival probabilities

**Figure 5:**
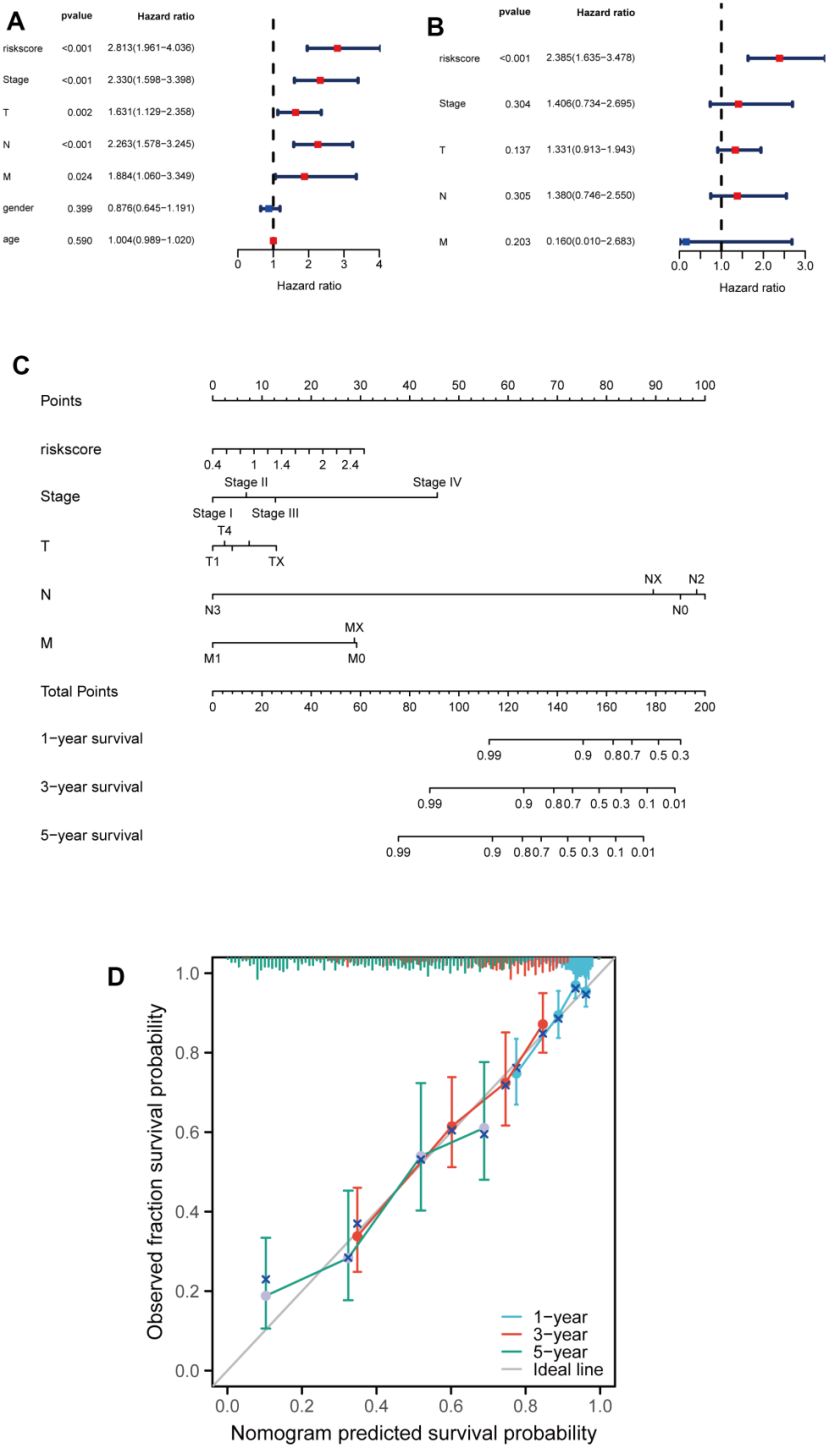
**Developing a nomogram for predicting LUAD patients' survival probabilities.** (**A**, **B**) Univariate and multivariate analysis of clinical features and riskscore on LUAD prognosis. (**C**) Nomogram for the prediction of the LUAD patients’ survival probability at 1, 3 and 5 years. (**D**) Calibration curves of TCGA dataset at 1, 3 and 5 years.

Subsequently, the univariable analysis and multivariable analysis based on the risk score, stage, T, N, M, age and gender were shown in Figure 5A, 5B, respectively. Interestingly, risk score is an independent prognostic factor (Figure 5A, 5B). Furthermore, to increase the clinical application's viability for LUAD patients, a nomogram was constructed based on the riskscore, stage, T, M, and N (Figure 5C). The total points were calculated by adding the above factors’ scores. Moreover, calibration plots showed good consistencies between the predicted curves and the actual curves of 1, 3, and 5 years (Figure 5D), suggesting the nomogram is beneficial for clinical prediction.

### Analysis of the correlation between the risk signature and genetic mutations

**Figure 6:**
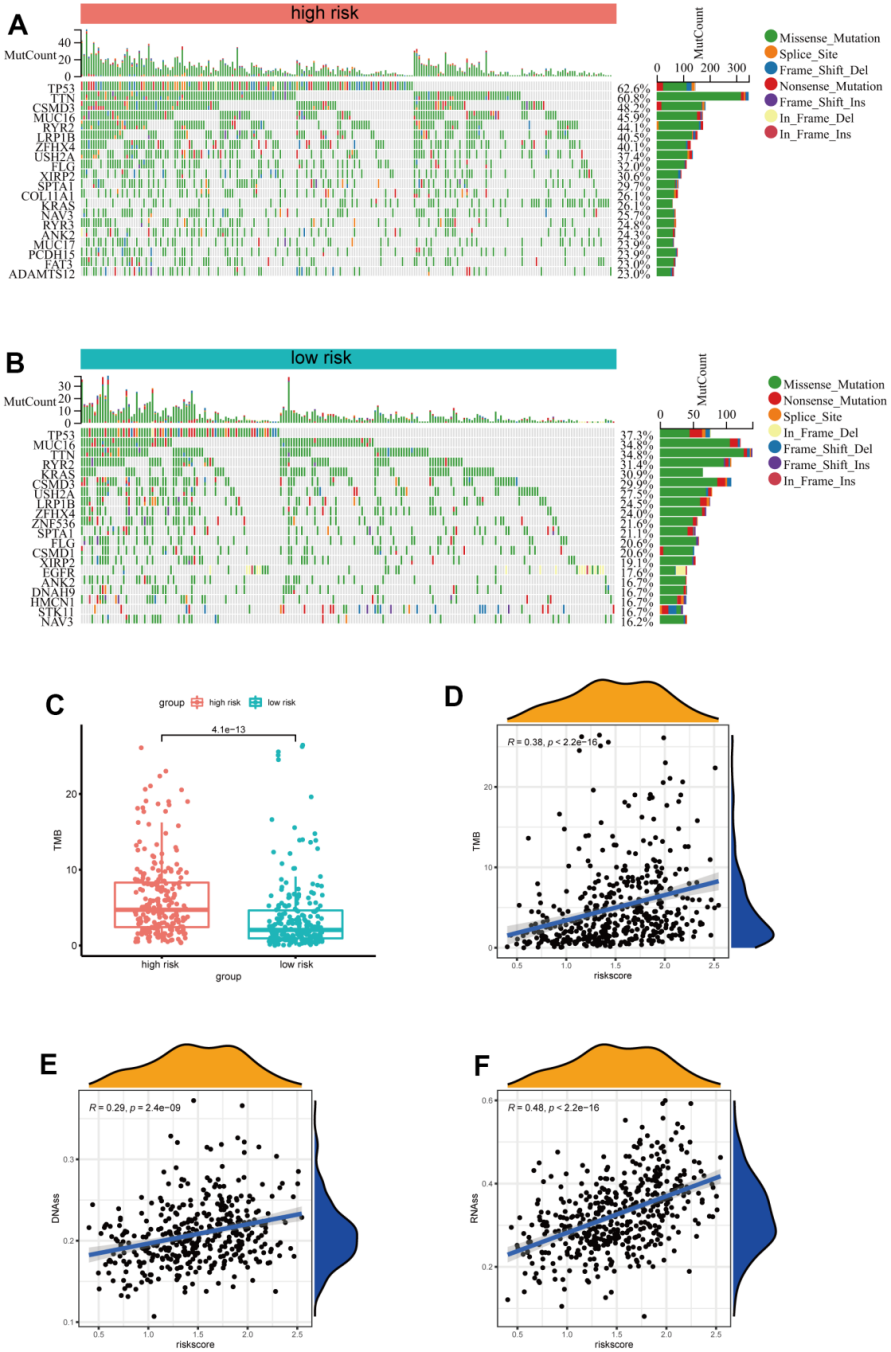
**Analysis of the correlation between the risk signature and genetic mutations.** (**A**) The top 20 driver genes with the highest alteration in the high-risk group. (**B**) The top 20 driver genes with the highest alteration in the low-risk group. (**C**) Box plot of the difference in risk score for patients with TMB. (**D**) Scatter plot of correlations between the TMB value and the risk score. (**E**, **F**) Scatter plot of correlations between the mRNAsi, mDNAsi and the risk score.

Subsequently, we further investigated the differences in somatic mutation distribution between low and high-risk scores in the TCGA set. As shown in the waterfall plot (Figure 6A, 6B), there are variations in the tumor mutational burden (TMB) of two subtypes, and the frequencies of TTN, CSMD3, MUC16, RYR2, LRB1P and ZFHX4 mutations in the high-risk group were considerably higher than those in the low-risk group. Furthermore, LUAD patients with high-risk scores had more TMB (Figure 6C) and the risk scores were positively correlated to the TMB values (R=0.48, p<0.001) (Figure 6D). Moreover, as indicated by DNA methylation levels, mDNAsi is a measure of stemness epigenetically, whereas mRNA expression is a measure of stemness transcriptomically [16, 17]. The risk scores of LUAD patients were positively correlated to the mRNA gene expression-based stemness index (mRNAsi) and DNA methylation-based stemness index (mDNAsi) (Figure 6E, 6F). These results indicate that the risk scores could predict the TMB and tumor stemness in the LUAD.

### Analysis of the risk signature, immune characteristics and therapy

**Figure 7:**
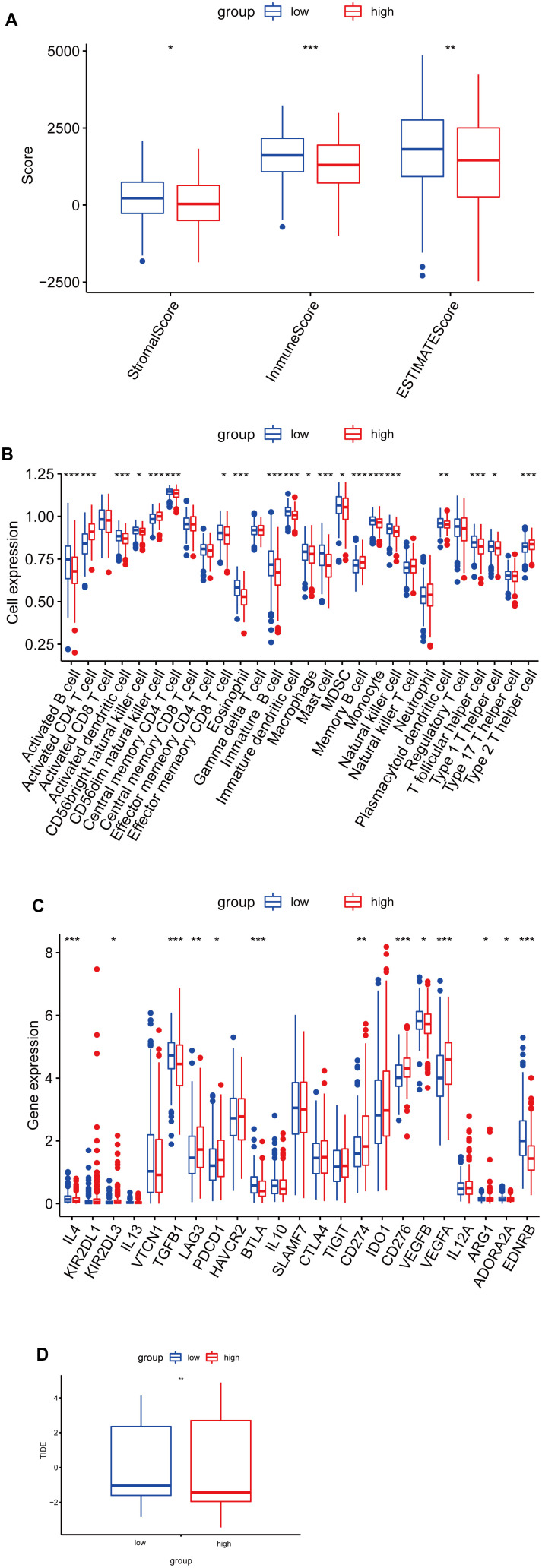
**Correlation analysis between risk score and immune infiltration in LUAD.** (**A**) Box plot of differences in ImmuneScore, StromalScore and EstimateScore between high- and low-risk groups. (**B**) Box plot of differences in immune cell infiltration in high- and low-risk groups. (**C**) Box plot of differences in checkpoint in high- and low-risk groups. (**D**) Box plot of differences in TIDE scores in high- and low-risk groups. *P < 0.05. **P < 0.01. ***P < 0.001.

To further explore the immune characteristics of the LUAD risk signature, we analyzed the relationship between the tumor microenvironment and risk score. As shown in Figure 7A, the high-risk group had lower stromal scores, immune scores, and estimate scores, compared to the low-risk score group. Furthermore, we analyzed the relationship between tumor-infiltrating immune cells and riskscore by ssGSEA, and found that the abundance of activated B cells, eosinophil, and mast cells in the high-risk group was significantly lower (Figure 7B). While the abundance of activated CD4 T cells in the high-risk group was significantly higher (Figure 7B). Furthermore, to investigate the sensitivity of LUAD patients to immune therapy, we analyzed the relationship between the immune checkpoints and risk score, and found that IL-4, TGFB1, BTLA, VEGFB, ADORA2A, and EDNRB expression levels were higher in the low-risk group, while KIR2DL3, LAG3, PDCD1, CD274, CD276, and VEGFA expression levels were higher in the high-risk group (Figure 7C). Moreover, the low-risk group had a higher TIDE score, indicating a poor immunotherapy response (Figure 7D).

### ARGs promote LUAD cell proliferation and migration

**Figure 8:**
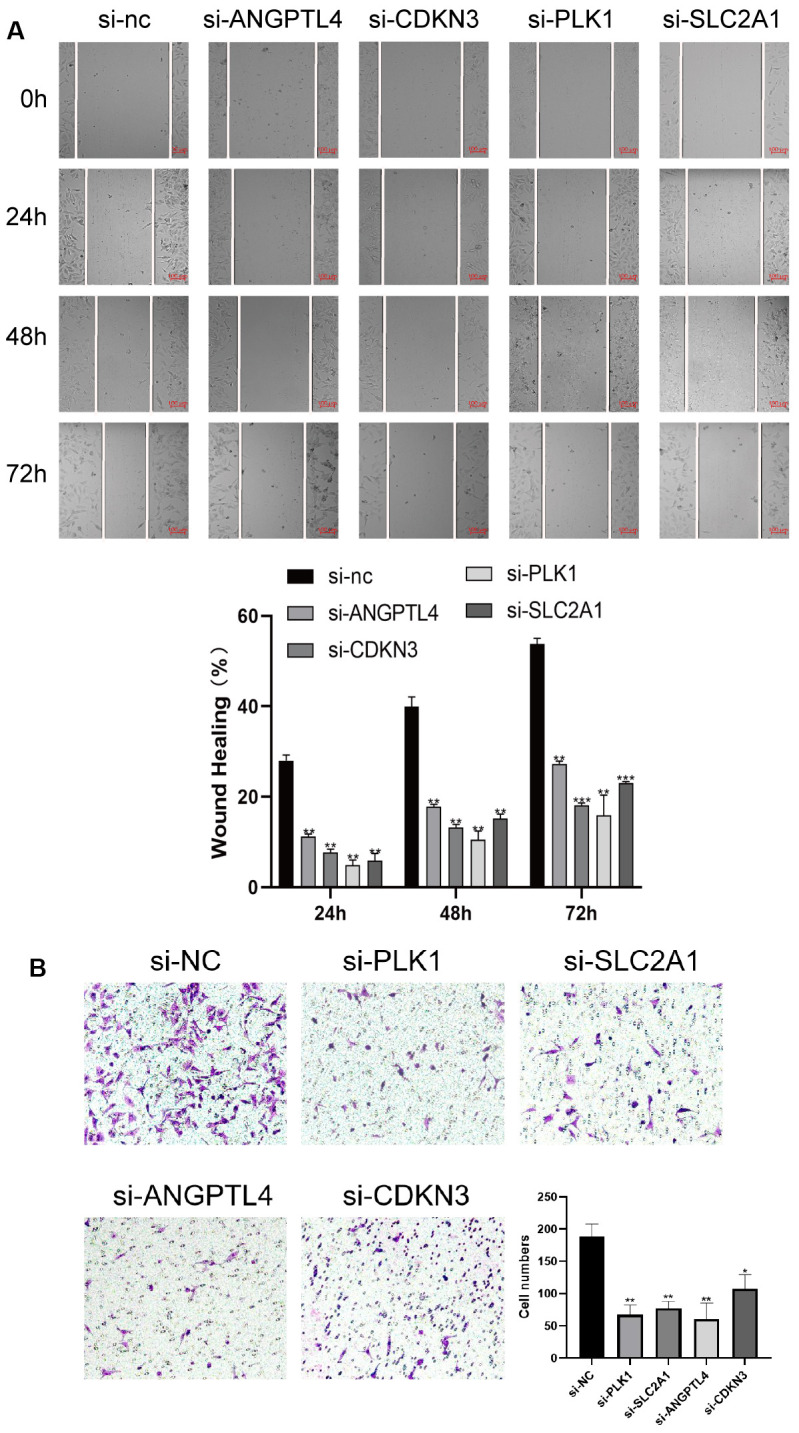
**ARGs promote LUAD cell proliferation and migration.** (**A**) Knockdown of 4 ARGs attenuated wound closure in A549 cells (n = 3). (**B**) Silencing 4 ARGs attenuated invasion in A549 cells. The error bars indicate the mean ± SD, and each experiment was repeated at least three times. *P < 0.05. **P < 0.01.

To verify the effects of the 4 ARGs on the function of LUAD cell, the 4 ARGs was knockdown by transfection with specific siRNAs in A549 cells, and the results of WB confirmed the of effectiveness of siRNAs (Supplementary Figure 2). Furthermore, we explored the effects of the 4 ARGs on LUAD cell proliferation and migration, and found that downregulation of PLK1, SLC2A1, ANGPTL4, or CDKN3 significantly inhibited the migration and invasion of A549 cells, respectively (Figure 8A, 8B).

### Knockdown of ARGs promotes anoikis of LUAD cell

**Figure 9:**
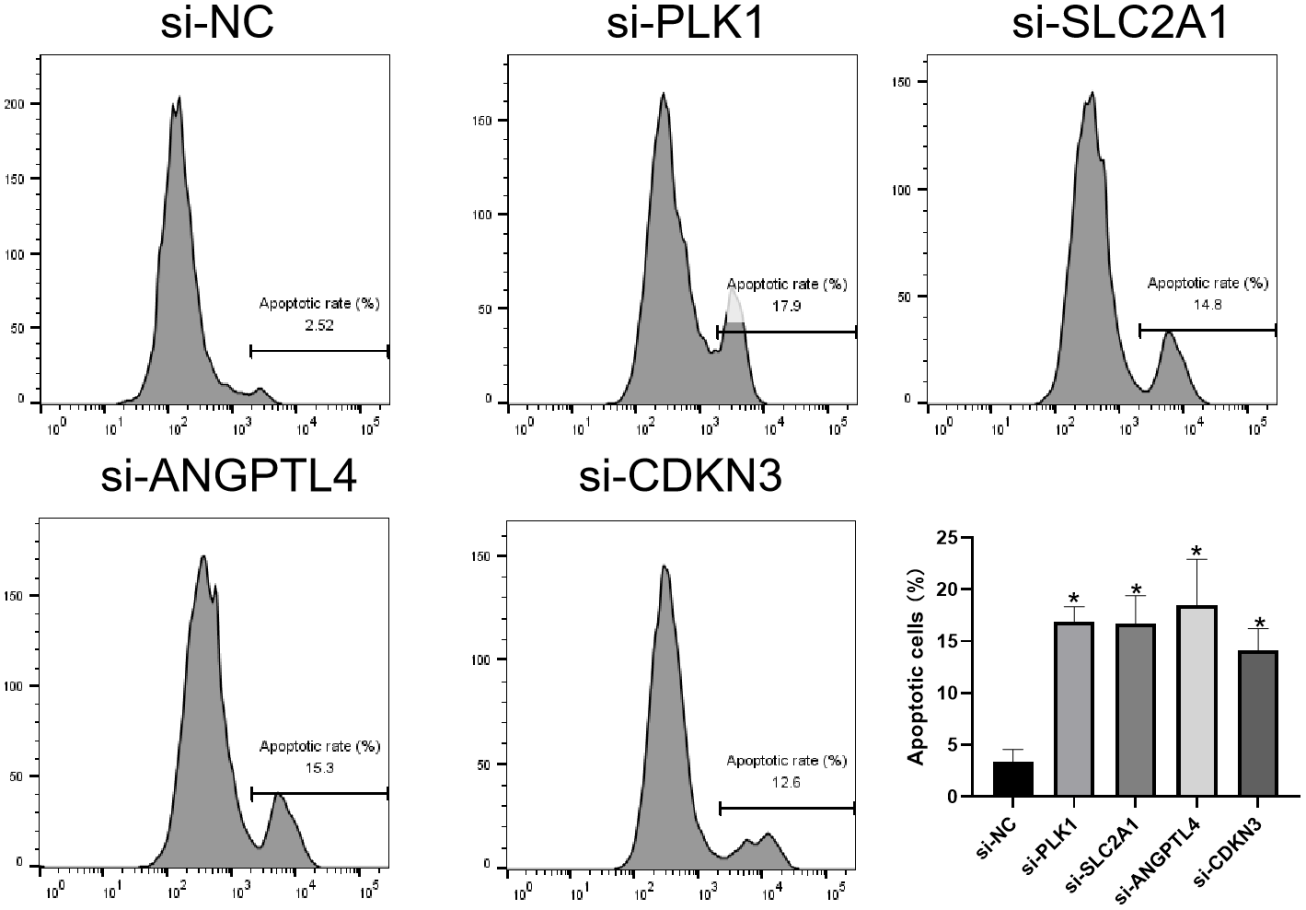
**Knockdown of ARGs promotes anoikis of LUAD cell.** Flow cytometry analysis of the apoptosis of A549 cells transfected with the indicated siRNAs. *P < 0.05.

Finally, we investigated the effects of 4 ARGs on the anoikis of LUAD cells via flow cytometry. As shown in Figure 9, knockdown of PLK1, SLC2A1, ANGPTL4, or CDKN3 significantly promotes anoikis of LUAD cells, respectively.

## DISCUSSION

Lung cancer is the primary killer of cancer patients worldwide and LUAD accounts for about 40% of all diagnosed cases [18]. Although molecular targeted anti-tumor drugs and immunotherapies have been wildly used to treat lung cancer [2], the survival time of LUAD patients is still not satisfying. Anoikis, a specific form of programmed cell death, is brought on by cell loss or improper adhesion [19, 20], and is closely associated with LUAD metastasis [21–23]. In this study, we screened four key ARGs (PLK1, SLC2A1, CDKN3, and ANGPTL4) by using two machine learning methods and constructed a riskscore prediction model to diagnose and predict LUAD patients, which may be useful in guiding clinical treatment for LUAD. The PLK1 plays a crucial role in regulating cellular mitosis, and is highly expressed many types of cancers, including LUAD [24, 25]. PLK1 is reported to enhance anoikis resistance via inhibiting β-catenin degradation in esophageal squamous cell carcinoma, and inhibition of PLK1 could trigger cell apoptosis to block LUAD progression [26–28]. SLC2A1, the glucose transporter, could encourage the growth, invasion, resistance to chemotherapy, and metastasis of cancer cells by controlling aerobic glycolysis [29, 30]. CDKN3, a cyclin-dependent kinase inhibitor, is usually high-expressed and regarded as a novel markers in several kinds of cancers [31–33]. CDKN3 promotes cancer growth via regulating cell cycle and DNA replication signaling [34], and inhibition of CDKN3 reduces cell proliferation, invasion and promotes apoptosis in cancer cells [35]. ANGPTL4 plays an important role in regulating aerobic glycolysis, the consumption of glutamine, and the oxidation of fatty acids [36]. ANGPTL4 also promotes tumor growth and enhances anoikis resistance in the scirrhous gastric cancer cells and the head and neck squamous carcinoma cells [37, 38].

Recently, Diao’s study identifies a 16 anoikis-related gene signature to predict prognosis and tumor microenvironment in LUAD [39]. In this study, we used two machine learning methods to avoid excessive variables, and screened four key ARGs (PLK1, SLC2A1, CDKN3, and ANGPTL4), three (PLK1, SLC2A1, and ANGPTL4) of which were also identified in Diao’s study. Whereas, we identified a novel anoikis-related gene-CDKN3, which is associated with poor survival in LUAD [14]. Besides, we further examined the four protein expressions in the clinical LUAD tissues, and found these proteins were all significantly highly expressed in the LUAD tissues. Furthermore, knockdown of PLK1, SLC2A1, CDKN3, or ANGPTL4 significantly inhibited the growth, invasion, and anoikis resistance in LUAD cells. Moreover, we found that the clinical features of LUAD, including T, N, and stages, were positively associated with the riskscore. In addition, the riskscore was more accurate at predicting prognosis of LUAD than other clinical data. LUAD patients with high-riskscores experienced shorter survival durations. A nomogram with integrated clinical characteristics and riskscore showed good accuracy. Overall, these results suggest that the 4 ARGs signature might be applied in clinical prognosis of LUAD.

Nowadays, the immune check-point inhibitors (ICIs) have been widely used for therapy in lung cancers [40], and the therapeutic effect is related to the expressions of immune check-points in tumors and TMB [41]. In this study, we found that LUAD patients with higher riskscore had higher levels of TMB, including TTN, CSMD3, MUC16, RYR2, LRB1P and ZFHX4 mutations, which would produce more neoantigens and improve T-cell recognition [42]. Titin (TTN) mutation is proved to act a beneficial role in lung squamous carcinoma [43], and is related to high immunogenicity and inflammatory tumor immune microenvironment (TIME) of LUAD [44]. MUC16 mutation is reported to be associated with genomic factors and response to ICI treatment in solid tumors [45]. Thus, LUAD patients with higher riskscore may be more suitable for ICB therapy, which needs further investigated. Besides, we also found the high-risk group of LUAD had lower stromal scores, immune scores, estimate scores, and IC50 of gemcitabine, compared to the low-risk score group. These results provided important information for the clinical precision treatment of LUAD.

This study has some limitations. Firstly, our analytical data are derived from public databases with relatively small sample sizes. Secondly, important analysis results need to be further validated with clinical samples. Thirdly, further large-scale basic studies can be carried out to verify the conclusions of this study.

In conclusion, the prognostic signature based on anoikis constructed in this study are helpful to predict the survival of LUAD and guide clinical treatment. Most importantly, LUAD patients in high-risk group are more suitable for immunotherapy and gemcitabine treatment. However, more experiments and clinical cases are needed to validate these findings.

## MATERIALS AND METHODS

### Data collection

The training set consisted of the transcriptomic profiles and clinical information of LUAD patients from the Tumor Genome Atlas (TCGA-LUAD, https://portal.gdc.cancer.gov/) database, including 456 samples. Based on their identification, the transcriptomic data was compared to the clinical data of the patients. The following criteria were used to filter the transcriptome data: (1) a histological diagnosis of LUAD; (2) a profile of relevant RNA expression; and (3) survival durations over 30 days. The validation set is from GSE50081, which included expression profiling of tumor tissues corresponding to 127 Stage I and II NSCLC cases collected at University Health Network. The information on the details of the LUAD patient in the TCGA-LUAD dataset and GSE50081 were shown in the Supplementary Table 3. A total of 347 anoikis-related genes were obtained from the GeneCard database (https://www.genecards.org/).

### Identification of anoikis-related DEGs (ARGs)

DEGs were found using the “limma” software [46]. As a cut-off value, we chose an adjusted P-value of 0.05 and a log2 foldchange (FC) greater than 1. The “ggpubr” and “ggplot2” packages were used for visualization of volcano maps and heat maps [47, 48].

### Functional enrichment analysis

The clusterProfiler tool [49] was used to compare biological topics among gene clusters. The BP, CC, and MF categories and signalling pathway enrichment analysis were performed using Kyoto Encyclopedia of Genes and Genomes (KEGG) and Gene Ontology (GO).

### Construction of a prognostic model for ARGs

According to the criteria, the training set was filtered from the TCGA dataset (n=456). The training set was then subjected to univariate Cox regression to identify ARGs that were related to survival (P-value<0.05). The most crucial feature genes were then screened using LASSO regression and random forest. For this investigation, the “glmnet” and “randomForest” packages were used [50, 51]. Two machine learning algorithms’ intersection was shown using a Venn diagram. Using multivariate Cox regression, we determined the LUAD riskscore as following: each potential prognostic gene had a regression coefficient that reflected it, and its expression value was given as Expi. The connection between clinical characteristics and riskscore in the TCGA, GEO dataset was visualized using the “ggplot2” and “ggpubr” packages. Based on the riskscore median, they were divided into two groups: high-risk group and low-risk group. The “survival” and “survminer” programs were used to examine Kaplan-Meier (K-M) survival curves in order to assess the prediction potential. The receiver operating curve (ROC) curve was created using the “pROC” R software [52]. By calculating the area under the ROC curve (AUC), we determined the diagnostic model’s classification ability.

### Immunohistochemical assay

Five LUAD tissues and five adjacent para-carcinoma tissues of LUAD patients were obtained from the Panyu Central Hospital. The study was approved by institutional ethics board of the Panyu Central Hospital (PYRC-2023-070). Immunohistochemical staining was performed, as previously described [52]. Sections were incubated with anti-ANGPTL4 rabbit primary antibodies (1:200; cat.no. 18374-1-AP; Proteintech), anti-PLK1 rabbit primary antibodies (1:200; cat.no. 10305-1-AP; Proteintech), anti-SLC2A1 rabbit primary antibodies (1:1000; cat.no. 21829-1-AP; Proteintech), anti-CDKN3 mouse primary antibodies (1:100; cat.no. D199341; Sangon Biotech) and secondary goat anti-rabbit IgG-HRP (1:200; cat. no.SA00001-2; Proteintech) antibodies.

### Construction of the nomogram of the ARGs signature with clinical features

Based on the clinical traits and the available ARGs signature, the nomogram was produced in TCGA using the “rms” package [53]. To assess the prediction performance, accuracy, and stability of this model, calibration curves for one, three, and five years were plotted.

### Analysis of tumor mutational burden, tumor microenvironment and drug sensitivity

The mutation frequencies and oncoplot waterfall plots for the two risk groups were examined and presented using the “maftools” software [54]. The new stemness indices, such as mRNAsi and mDNAsi, produced by the OCLR machine-learning algorithm analyse the relationship with riskscore through spearman correlation analysis [55]. To explore the expression of 28 immune cell types, single-sample gene set enrichment analysis (ssGSEA) was used to analyze each sample in high- and low-risk groups. The immunescore, stromalscore, and tumorpurity of each sample were determined using the expression data (ESTIMATE) tool and the “estimate” package [56, 57]. To forecast each patient’s sensitivity to different medications, the “pRRophetic” software was employed [58]. To explore the underlying immunotherapy response of patients, tumor immune dysfunction and exclusion (TIDE) scores were examined.

### Cell culture and transfection

The cell lines present in this study were obtained from the Procell Life Science and Technology Co., Ltd (Wuhan, China). LUAD line A549 cells were cultured in high glucosecontaining DMEM supplemented with 10% fetal bovine serum in 95% humidified air and 5% CO_2_ at 37° C.

For siRNA transfection, A549 cells were plated in 6-well plates at 60-70% confluence, and then transfected with the mixture of lipofectamine 2000 (Invitrogen, Thermo Fisher Scientific, Shanghai, China) and 200 ng siRNA. siRNA against ANGPTL4 (si-ANGPTL4, GCG AAU UCA GCA UCU GCA A), SLC2A1 (si-SLC2A1, GUG UUU AGA ACA GCG UCU A), CDKN3 (si-CDKN3, AGA ACU AAA GAG CUG UGG UAU) and PLK1 (si-PLK1, CGA GGU GCU GAG CAA GAA A) and their negative control (scramble, UUC UCC GAA CGU GUC ACG U) were purchased from RiboBio Co., Ltd (Guangzhou, China). 24 h after transfection, the cells were used for further experiments.

### Real-time PCR testing (qRT-PCR)

Using the FastPure Cell/Tissue Total RNA Isolation Kit, total RNA was isolated from the cells (Vazyme, Nanjing, China). Reverse transcription was carried out using HiScript RT supermix for qPCR (Vazyme, Nanjing, China). The expression levels of the genes were assessed using ChamQ Universal SYBR qPCR Master Mix (Vazyme, Nanjing, China) via ABI 7500 Fast Real-Time System. The reaction mixtures underwent 35 cycles of 95° C for 15 seconds, 60° C for 30 seconds, and 95° C for 15 seconds after being incubated at 95° C for 2 minutes. The relative expression of mRNA was normalized using the 2-^ΔΔCt^ method relative to GAPDH. Primer sequences were as follows: ANGPTL4, 5’-GTCCACCGACCTCCCGTTA-3’ (forward) and 5’-CCTCATGGTCTAGGTGCTTGT-3’ (reverse); CDKN3, 5’-TCCGGGGCAATACAGACCAT-3’ (forward) and 5’-GCAGCTAATTTGTCCCGAAACTC-3’ (reverse); PLK1, 5’-CCTGCACCGAAACCGAGTTAT-3’ (forward) and 5’-CCGTCATATTCGACTTTGGTTGC-3’ (reverse); SLC2A1, 5’-TCTGGCATCAACGCTGTCTTC-3’ (forward) and 5’-CGATACCGGAGCCAATGGT-3’ (reverse); GAPDH, 5’-GATCATCAGCAATGCCTCCT-3’ (forward), reverse: 5’-TTCAGCTCAGGGATGACCTT-3’ (reverse).

### Scratch wound healing assay

4 × 10^5^ A549 cells were plated into a 12-well plate. When the cells covered 90% of the plate's bottom area, a 20μL sterile pipette tip was used to scratch the plate vertically. To ensure the visual field was clear for photography, the culture medium in the plate was removed, gently washed with PBS, and then the cell debris was rinsed off. Microscopy was used to track cell movement after 24 hours.

### Transwell assay

At the upper transwell chamber insert, A549 cells in the logarithmic growth phase were planted at a density of 3 × 10^4^ cells per well. Serum-free cell culture media was placed in the upper chamber of a 24-well plate, and 10% FBS complete medium was placed in the lower chamber. For 24 hours, the culture was maintained. To count the migrating cells, the medium was discarded and stained with a crystal violet solution.

### Anoikis assay

An ethanol solution containing 12 mg/ml of Poly-HEMA (Sigma-Aldrich, St Louis, USA), a non-adhesive substrate, was applied to each well of 6-well plates, and the solution was then allowed to evaporate to dryness at room temperature. Following washing with PBS for twice, the plates were then plated with the transfected cells. Culture for 12 hours, and Annexin V-FITC labeling was used to examine cell apoptosis (Vazyme, Nanjing, China).

### Statistical analysis

R software (version R-4.1.0) and GraphPad Prism 8.0.2 were used for all statistical analyses. The Wilcoxon test and Kruskal-Wallis test were used for continuous variable analysis between groups. For the bivariate correlation analysis, Spearman correlation analysis was performed. The significance level is denoted as follows: *P < 0.05, **P < 0.01, ***P < 0.001.

### Data availability

The data analyzed in the present study are publicly available on the TCGA and GEO database. The datasets used and/or analyzed during the current study are available from the corresponding author on reasonable request.

## Supplementary Material

Supplementary Figures

Supplementary Table 1

Supplementary Table 2

Supplementary Table 3
