# Supplementary material for: Identification of crucial anoikis-related genes as novel biomarkers and potential therapeutic targets for lung adenocarcinoma via bioinformatic analysis and experimental verification
Source: Aging (Albany NY). 2024 Feb 9;16(3):2887–907. doi: 10.18632/aging.205521 (PMC10911345; doi:10.18632/aging.205521)
Supplement: Supplementary Figures [file aging-16-205521-s001.pdf]

SUPPLEMENTARY FIGURES

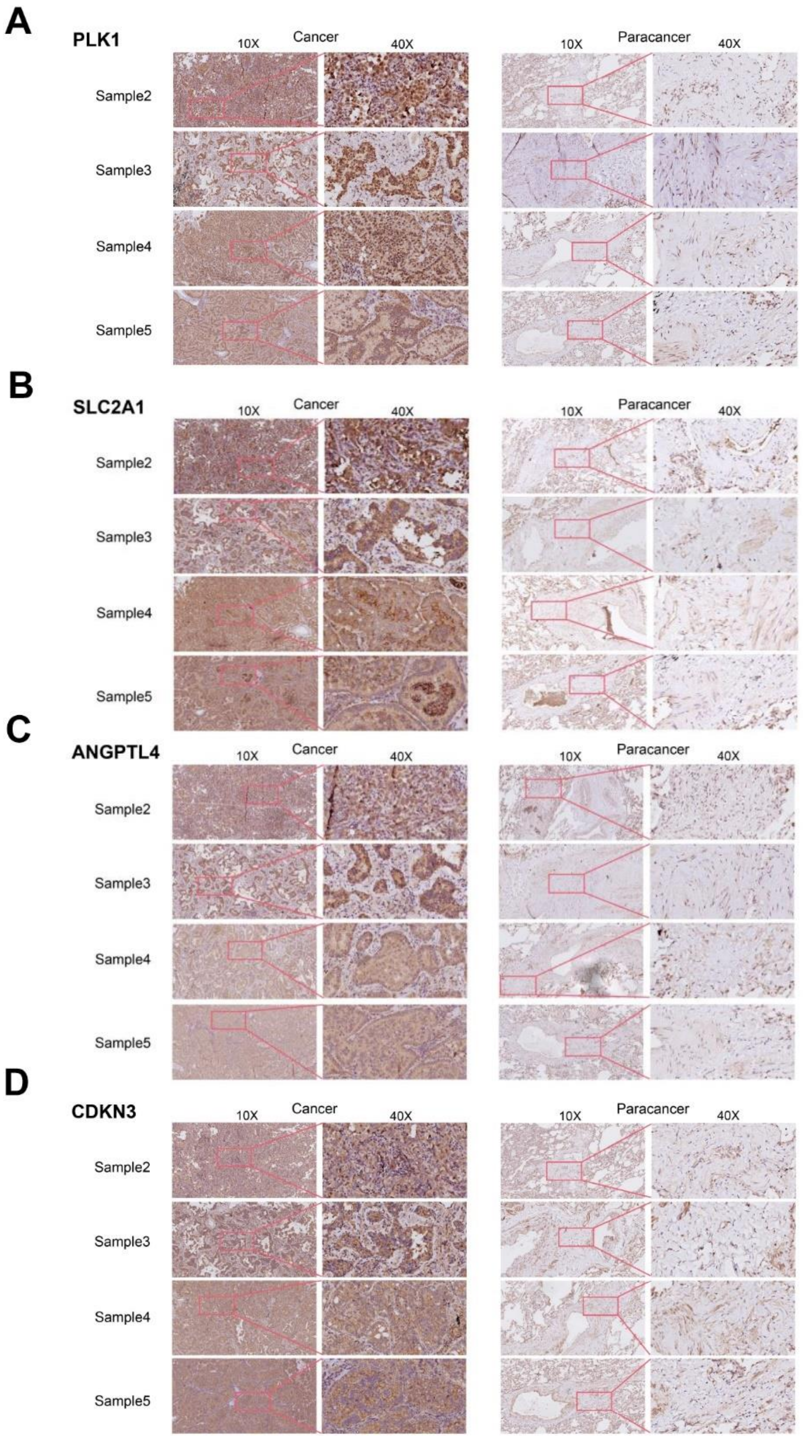

Supplementary Figure 1. Immunohistochemical staining of four ARGs in cancer tissues and para-cancer tissues from clinical LUAD patients. (**A**) Immunohistochemical staining of PLK1. (**B**) Immunohistochemical staining of SLC2A1. (**C**) Immunohistochemical staining of ANGPTL4. (**D**) Immunohistochemical staining of CDKN3.

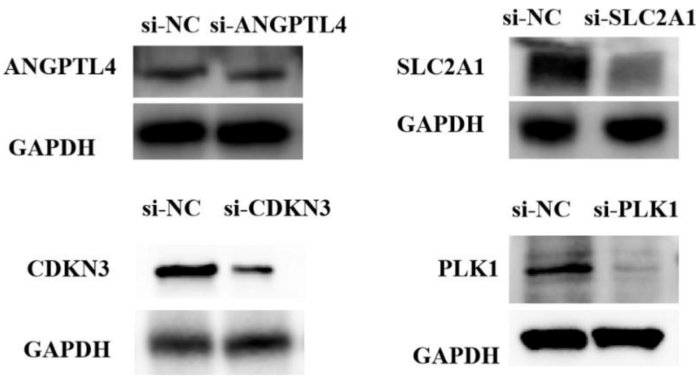

Supplementary Figure 2. Western blotting analysis of the indicated protein expression in A549 cells transfected with the indicated siRNAs.
