# Supplementary material for: Identification of crucial anoikis-related genes as novel biomarkers and potential therapeutic targets for lung adenocarcinoma via bioinformatic analysis and experimental verification
Source: Aging (Albany NY). 2024 Feb 9;16(3):2887–907. doi: 10.18632/aging.205521 (PMC10911345; doi:10.18632/aging.205521)
Supplement: Supplementary Table 3 [file aging-16-205521-s004.pdf]

SUPPLEMENTARY TABLE

Supplementary Table 3. The information on the details of the LUAD patient in the TCGA-LUAD dataset and GSE50081.

Characteristics	Training set (TCGA-LUAD,n=456)	Validation set (GSE50081, n=127)
Age		
< 65	203(44.5%)	40(31.5%)
≥ 65	243(53.3%)	87(68.5%)
NA	10(2.2%)	NA
Gender		
Female	250(54.8%)	62(48.8%)
Male	206(45.2%)	65(51.2%)
Race		
White	374(82.0%)	NA
Non-white	59(12.9%)	NA
Unknown	23(5.0%)	NA
Ethnicity		
Hispanic or Latino	7(1.5%)	NA
Non-Hispanic or Latino	370(81.1%)	NA
Unknown	79(17.3%)	NA
Tumor stage		
Stage I	249(54.6%)	92(72.4%)
Stage II	107(23.5%)	35(27.6%)
Stage III	71(15.6%)	NA
Stage IV	22(4.8%)	NA
Unknown	7(1.5%)	NA
T classification		
T1	160(35.1%)	43(33.9%)
T2	242(53.1%)	82(64.6%)
T3	37(8.1%)	2(1.6%)
T4	14(3.1%)	NA
Unknown	3(0.7%)	NA
Smoking history		
Ever	319(70.0%)	92(72.4%)
Never	120(26.3%)	23(18.1%)
Unknown	17(3.7%)	12(9.4%)
Vital status		
Alive	168(36.8%)	76(59.8%)
Dead	288(63.2%)	51(40.2%)
